# Determinants of Neural Plastic Changes Induced by Motor Practice

**DOI:** 10.3389/fnhum.2021.613867

**Published:** 2021-01-28

**Authors:** Wen Dai, Kento Nakagawa, Tsuyoshi Nakajima, Kazuyuki Kanosue

**Affiliations:** ^1^Graduate School of Sport Sciences, Waseda University, Saitama, Japan; ^2^Faculty of Sport Sciences, Waseda University, Saitama, Japan; ^3^Department of Integrative Physiology, Kyorin University School of Medicine, Tokyo, Japan

**Keywords:** motor practice, transcranial magnetic stimulation, paired associative stimulation, primary motor cortex, goal setting, task-dependent plasticity

## Abstract

Short-term motor practice leads to plasticity in the primary motor cortex (M1). The purpose of this study is to investigate the factors that determine the increase in corticospinal tract (CST) excitability after motor practice, with special focus on two factors; “the level of muscle activity” and “the presence/absence of a goal of keeping the activity level constant.” Fifteen healthy subjects performed four types of rapid thumb adduction in separate sessions. In the “comfortable task” (C) and “forceful task” (F), the subjects adducted their thumb using comfortable and strong forces. In the “comfortable with a goal task” (CG) and “forceful with a goal task” (FG), subjects controlled the muscle activity at the same level as in the C and F, respectively, by adjusting the peak electromyographic amplitude within the target ranges. Paired associative stimulation (PAS), which combines peripheral nerve (median nerve) stimulation and transcranial magnetic stimulation (TMS), with an inter-stimulus interval of 25 ms (PAS25) was also done. Before and after the motor tasks and PAS25, TMS was applied to the M1. None of the four tasks showed any temporary changes in behavior, meaning no learning occurred. Motor-evoked potential (MEP) amplitude increased only after the FG and it exhibited a positive correlation with the MEP increase after PAS25, suggesting that FG and PAS25 share at least similar plasticity mechanisms in the M1. Resting motor threshold (RMT) decreased only after FG, suggesting that FG would also be associated with the membrane depolarization of M1 neurons. These results suggest task-dependent plasticity from the synergistic effect of forceful muscle activity and of setting a goal of keeping the activity level constant.

## Introduction

The primary motor cortex (M1) plays an important role in controlling voluntary movements. It shows plasticity after motor practice, either short-term (Bütefisch et al., 2000) or long-term (Dai et al., 2016). The motor cortex in mammals exhibits a high degree of motor-dependent plasticity: with motor practice, horizontal neuron connections in the M1 are enhanced, and the M1 presents an obvious mapping reorganization (Sanes and Donoghue, 2000). Animal studies have suggested that motor practice could strengthen the synaptic connections of cortical neurons in the M1 (Rioult-Pedotti et al., 2000). Synaptic plasticity in the M1 is considered as the most probable mechanism to explain this excitability change with motor learning (Sanes, 2003). Since the M1 plasticity cannot be directly observed in human studies, transcranial magnetic stimulation (TMS) is widely used to investigate the M1 or corticospinal tract (CST) plasticity. An increase in motor-evoked potential (MEP) amplitude, regarded as facilitation of CST excitability, is consistently observed after motor practice, such as repeated rapid thumb abduction; this increase lasts for tens of minutes to days (Ziemann et al., 2004; Rosenkranz et al., 2007).

As a research model of synaptic plasticity in the M1, long-term potentiation (LTP) has been extensively studied. LTP relies on the process of synaptic modification with an increase in synaptic transmission that persists for minutes, days, or even weeks (Cooke and Bliss, 2006). Instead of directly assessing LTP plasticity after motor practice, non-invasive brain stimulation (NIBS) techniques are widely used in human studies; these include repetitive transcranial magnetic stimulation (rTMS), theta-burst stimulation (TBS; modified rTMS), transcranial direct current stimulation (tDCS), and paired associative stimulation (PAS). iTBS, tDCS, and PAS have been demonstrated to induce neuroplasticity, observed as changes in cortical excitability that lasted approximately 90 to 120 min (Nathan et al., 2011). These plasticity induced by NIBS techniques share major properties with LTP (Cooke and Bliss, 2006) in that both show rapid evolution, persistence, reversibility, and dependence on the mediation of N-methyl-D-aspartic (NMDA) receptors; thus, they are termed “LTP-like plasticity” (Ridding and Ziemann, 2010).

Among them, PAS, peripheral nerve stimulation combined with TMS to the M1, primarily induces the glutamatergic plasticity of specific neurons in somatosensory-motor cortex connections (Stefan et al., 2002). Several studies showed that induction by PAS was more specific in the M1 than induction by other NIBS techniques (Di Lazzaro et al., 2011; Player et al., 2012; Vallence et al., 2013). Considering the transmission time of afferent signals produced by peripheral stimulations, a peripheral stimulation applied 25 ms before TMS (PAS25) reaches the M1 at almost the same time as the TMS, and this induces synaptic plasticity in the M1 with increased MEPs (Stefan et al., 2000, 2002). Since there is no change in F-wave and brainstem stimulation-evoked potential after PAS25, PAS25 is thought to induce LTP-like plasticity occurring only at the level of the motor cortex (Stefan et al., 2000).

To elucidate whether the mechanisms of plasticity in the M1 after motor practice is shared with LTP, motor-practice-induced plasticity and PAS-induced plasticity were compared in many studies (Ziemann et al., 2004; Rosenkranz et al., 2007; Vallence et al., 2013; Hamada et al., 2014). However, there remains contradiction among studies: while the plasticity induced by motor learning was considered functionally similar to PAS-induced LTP-like plasticity in some studies (Ziemann et al., 2004; Stefan et al., 2006; Rosenkranz et al., 2007; Avanzino et al., 2015), another study proposed that the mechanisms of these two would only partially overlap because there was no correlation between MEP amplitude changes by motor practice and PAS (Vallence et al., 2013). One reason for this contradiction could be that there were differences in motor tasks among those studies.

However, the mechanism by which differences in motor tasks affect motor cortical or corticospinal plasticity has not yet been systematically investigated. In this study, we focused on the interaction of two motor task factors, “the level of muscle activity” and “the presence/absence of a goal of keeping the activity level constant.” These two factors have been shown to affect the CST excitability. First, the MEP amplitude significantly increased after wrist flexion tasks with 70% of maximum voluntary contraction (MVC), while there was no increase after the same task with 10% MVC (Perez and Cohen, 2009). Meanwhile, after five wrist extension tasks with different isometric muscle strengths below 50% MVC, there was no significant difference in the change in MEP amplitudes among tasks (Samii et al., 1996). Second, setting a clear goal of motor practice, which would require more attention, produced a significant increase in MEP amplitude, while no significant changes in MEP were produced after motor practice without a goal (Smyth et al., 2010). The purpose of this study is to investigate how motor practice with various combinations of the two factors, “the level of muscle activity” and “the presence/absence of a goal of keeping the activity level constant,” affect the change in the CST excitability, and how the factors interact with each other. Therefore, using the motor practice of thumb adduction as an experimental model, we set two levels of muscle activity, comfortable and forceful; at each level subjects did two tasks, one with and one without the goal of keeping the activity level constant.

We analyzed whether each of the four tasks could induce an increase in MEP amplitude and examined whether LTP-like synaptic plasticity was associated with the observed increase in MEP amplitude by analyzing the correlation between the MEP changes induced by motor practice and the MEP changes induced by PAS25. It was hypothesized that an increase in MEP amplitude occurs after a task that requires both forceful muscle activity and setting a goal of keeping the activity level constant, and this MEP increase has a positive correlation with the increase in MEP amplitudes after PAS25, which suggests that they share at least similar mechanisms in the M1 (Vallence et al., 2013).

## Materials and Methods

### Subjects

Eighteen healthy subjects (13 males and five females, two left-handed, aged 24.9 ± 2.2 years) participated in this study. Handedness was assessed using the Edinburgh handedness inventory (Oldfield, 1971). No subject was taking neuroactive medication before or during the experiments. All subjects provided written informed consent following the Declaration of Helsinki. The experimental protocol was approved by the Human Research Ethics Committee of Waseda University (2018-040).

### Electromyographic (EMG) Recording

EMG activity was recorded with Ag-AgCl surface electrodes (1 cm diameter) from the dominant-hand abductor pollicis brevis (APB) muscle. The signal was amplified and band-pass filtered between 5 Hz and 3 kHz (MEB-2216, Nihon Koden, Japan), digitized at 3,000 Hz by an analog-to-digital interface (Micro 1401, Cambridge Electronics Design, Cambridge, UK), and stored in a computer for offline analysis.

### Motor Task

The motor tasks were based on tasks used in previous studies (Ziemann et al., 2004; Jung and Ziemann, 2009; Delvendahl et al., 2012; Vallence et al., 2013). Before every task, EMG activity during a 3 s MVC was measured three times. Subjects were asked to perform rapid thumb adduction 90 times in response to a metronome tone at 0.2 Hz. Subjects were instructed to perform the thumb adduction as fast as possible during each trial and then to return their thumb to the neutral rest position to prepare for the subsequent trial.

“Comfortable” and “forceful” muscle activity were selected as the first two task requirements. In the “comfortable task” (C) and the “forceful task” (F), the subjects were required to adduct their thumb using comfortable and strong forces, respectively, at levels determined by the subjects themselves. For the F, however, subjects were asked to practice generating forceful muscle activity for several trials (less than 30 s each) before the main trials. If the peak EMG amplitude during practice was less than 70% MVC, verbal encouragement was given to prompt them to greater effort. In the main F trials, subjects were instructed to exert an effort of similar strength as in practice, but we did not provide any verbal encouragement even if the EMG amplitude fell below 70% MVC. We instructed subjects to concentrate on their thumb and look at a computer screen during C and F which showed the EMG activity in real-time.

**Figure 1:**
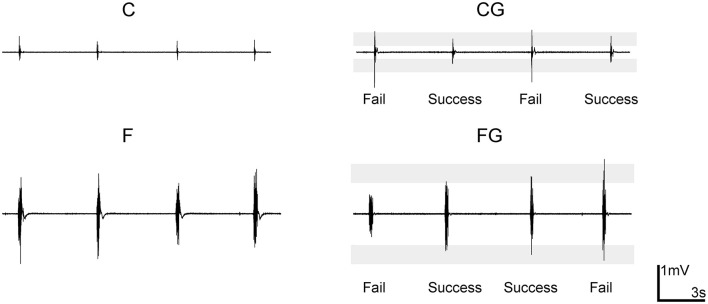
Example recordings of electromyogram (EMG) level in motor tasks. Data from a representative subject (male, right-handed). The shading of two controlling tasks comfortable with a goal task and forceful with a goal task (CG and FG) indicates the target range. “Success” and “fail” indicate whether the muscle activity of that trial was inside the target range.

“With” and “without” a goal of keeping the activity level constant were selected as the second two task requirements. The C and F described above lacked a specific goal to control muscle activity; in contrast, the “comfortable with a goal task” (CG) and “forceful with a goal task” (FG) included controlling the muscle activity at the same level as in the C and F, respectively, by controlling the peak EMG so that it fell within target ranges (Figure 1). The target ranges were determined from preliminary experiments.

The preliminary experiments were done after subjects performed the C and F tasks. Nine subjects who completed both the C and F tasks participated in this preliminary experiment. Subjects were required to control the peak EMG activity of thumb adduction and could see their EMG activity with a target range, enabling them to adjust their muscle activation in the subsequent trial. The level of EMG activity was pre-determined as an average of the C and F tasks, M_C_ and M_F_, respectively. The target range was set as one of M_C_/M_F_ ± 1/3 SD, 1/2 SD, 1 SD, or 1.5 SD (SD = SD_C_ for C, and SD_F_ for F). In total eight preliminary experiments (four experiments at each level of muscle activity) were done on separate days. At least 48 h elapsed between any two preliminary experiments to avoid an interaction effect between motor tasks. From the results obtained from this series of preliminary experiments, the target ranges of CG and FG in the main experiments were chosen as Mc ± 1 SDc and M_F_ ± 1.5 SD_F_, respectively, so that the success rates of CG and FG became comparable. The success rate was calculated as [(number of trials in which peak EMG fell in the target range)/90] ×100%. In the main CG and FG experiment, subjects were also allowed to see their target range and their EMG performance, enabling them to adjust their muscle activation in the subsequent trial.

### TMS

One Magstim 200 stimulator (Magstim Ltd., UK) and a figure-eight-shaped coil (outside diameter of each loop was 9.5 cm) were used to deliver TMS to the M1 contralateral to the target muscle. The handle of the coil pointed backward at 30–45° from the mid-sagittal line (Werhahn et al., 1994). The current was induced in the posterior-anterior direction in the brain, approximately perpendicular to the central sulcus (Kaneko et al., 1996). The TMS coil was placed at the optimal position where stimulation with slight suprathreshold intensity produced the largest MEP in the target muscle.

To assess corticospinal excitability, the baseline TMS intensity to be used throughout the experiment was determined for each subject as the minimum stimulator output that generated an average MEP amplitude of 0.5–1 mV in 10 trials when the target muscle was completely relaxed. Resting motor threshold (RMT) was also determined before and after the experiment to explore the motor threshold at resting muscle state. RMT was defined as the minimum stimulator output that generated MEPs of more than 50 μV in at least five out of 10 trials when the target muscle was completely relaxed (Dai et al., 2016). Each test was recorded in random order with an inter-trial interval of 5 s.

### PAS

In the PAS protocol, 90 pairs of peripheral nerve and TMS stimulations were conducted at 0.2 Hz. The total number of trials and the frequency of PAS25 were the same as those in motor tasks to equalize the intervention parameters. Peripheral nerve stimulation was applied to the median nerve of the dominant wrist using an electrical current stimulator (SS-104J, Nihon Koden, Japan) through bipolar surface electrodes (1 cm diameter) with the cathode proximal. Stimuli were square waves with a pulse width of 200 μs. The stimulus intensity was set at 300% of the perceptual threshold, the lowest stimulus intensity felt by each subject (Stefan et al., 2000; Wolters et al., 2003). TMS to the cortical area of the APB in the contralateral M1 followed the peripheral nerve stimulation with a 25 ms interstimulus interval (PAS25) and TMS stimulations used the baseline MEP intensity. Subjects were asked to relax their target muscle during the PAS protocol. A computer screen that displayed background EMG amplitude (b.EMG) in real-time was shown to the experimenter and subjects. If the subjects voluntarily contracted the target muscle during the PAS intervention, a verbal reminder was given to the subject to relax their muscle immediately. Additionally, to maintain a constant state of attention and visual inputs, we also instructed subjects to concentrate on their thumb and look at the computer screen so they could monitor their b.EMG in real-time during the PAS intervention (Stefan et al., 2004).

In the preliminary experiments in advance of the main PAS intervention, we tested the PAS25 protocol as noted above to recruit only PAS responders as subjects (Cheeran et al., 2008), considering the large inter-individual variability of the PAS effect (López-Alonso et al., 2014). Non-responders did not participate in the subsequent main experiments. A grand average analysis was conducted to screen “PAS responders”: if the average MEP amplitude at 30 min after PAS25 was bigger than that at baseline, that subject would be classified as a “PAS responder” (Müller-Dahlhaus et al., 2008; López-Alonso et al., 2014). As a result, three subjects (one female, no left-handed) were excluded, and analysis was conducted for the remaining 15 subjects.

### Main Experimental Protocol

Five protocols with interventions of PAS25, C, F, CG, and FG were performed. All subjects completed all five protocols. At least 1 week elapsed between each of the five protocols to avoid an interaction effect between motor tasks and PAS (Ziemann et al., 2004). Additionally, at least 1 month elapsed between the preliminary and the main experiments. MVC, baseline values of stimulus intensity, MEP amplitude, and RMT were measured before each intervention for each subject. MEPs after interventions were obtained with the same stimulus intensity, number, and frequency as the baseline measurement, and at 5, 10, 15, 20, 25, and 30 min after the task (T5, T10,…, T30) to evaluate changes in CST excitability. RMT were also measured again after all MEP measurements at T30. The b.EMG was shown on a computer screen in real-time to the experimenter for checking whether the subjects relaxed their target muscle during measurement of RMT and MEPs.

The order of C and F were randomly arranged. However, the C was always conducted before the CG and the F was always conducted before the FG, to define the target range in the CG and FG.

### Statistics

Values were expressed as average ± standard error (SE). Muscle activity recorded during 90 trials for each task was evaluated using the average of peak-to-peak EMG amplitude, which was expressed as the value normalized with the MVC value. MEP amplitude was measured as the peak-to-peak value. For each subject and in each task, the MEP amplitude at each time point (T5–T30) was normalized with the pre-intervention MEP amplitude (baseline).

One-way repeated-measures analysis of variance (rmANOVA) was used to test for the differences among four motor tasks in MVC, and five interventions in baseline MEP amplitude, stimulus intensity, and RMT before the intervention. Two-tailed paired-sample *t*-tests were used to test the differences between pre- and 30 min post-intervention RMT of five protocols.

The success rate of the CG and FG was calculated as [(number of trials in which peak EMG was inside the target range)/90] ×100%. For the CG and FG, we also calculated the degree of error as the absolute difference between the peak EMG and the target value (M_C_ in CG and M_F_ in FG). The degree of error was normalized with the target value. In each behavioral index, we created epochs by binning 10 consecutive movements. In total nine epochs (i.e., 90 trials) for each task were set. One-way rmANOVA with factor “epoch” was used to test the temporary changes in the peak amplitude of muscle activity, as well as success rate and degree of error in each motor task to determine whether the level of muscle activity changed or, separately, whether motor performance improved (i.e., motor learning occurred). Two-way rmANOVA with factors “muscle activity (comfortable and forceful muscle activity)” and “goal-setting (with and without a goal of keeping the activity level constant)” was used to test for the differences in average peak EMG activity among four motor tasks. Two-tailed paired-samples *t*-tests were used to compare the difference in the average success rate and degree of error between the CG and FG.

The Friedman test was performed to test for temporary changes (from baseline to 30 min) in MEP amplitude for each task. The Wilcoxon test was used in *post hoc* analysis when the Friedman test detected a significant effect. To observe whether MEP amplitude changed by intervention, the average MEP amplitude of all time points after the intervention (from T5 to T30) was compared to 100% by two-tailed one-sample *t*-tests with Bonferroni’s correction. One-way rmANOVA was used to compare the differences between the five interventions. We used the Greenhouse–Geisser correction to adjust for violations of sphericity, if necessary. Bonferroni’s correction for multiple comparisons was used in *post hoc* analysis when ANOVA detected a significant main effect or interaction.

A linear regression analysis was performed to evaluate the relationship between the average MEP amplitudes of all time points after the intervention in each motor task and PAS25. The analysis used MEP amplitudes of PAS25 as the independent variable and MEP amplitudes of motor tasks as the dependent variables.

The significance threshold was set at *P* < 0.05. SPSS version 17.0 software (IBM, Armonk, NY, USA) was used for statistical analysis.

## Results

Table 1 shows MVC before four motor tasks; baseline MEP amplitude, stimulus intensity, and RMT before five interventions; and RMT changes after interventions. There were no significant main effects among four motor tasks in MVC (*F*_(3,42)_ = 0.99, *P* = 0.405). Baseline MEP amplitude, stimulus intensity, and RMT did not show main effects among five interventions (baseline MEP amplitude: *F*_(2.27,31.82)_ = 2.13, *P* = 0.090; baseline intensity: *F*_(4,56)_ = 2.09, *P* = 0.095; RMT: *F*_(4,56)_ = 0.19, *P* = 0.941). RMT significantly decreased 30 min after intervention only in the FG (Bonferroni’s correction, significance threshold was set at *P* < 0.01, i.e., 0.05/5 interventions = 0.01. PAS25: *t* = −0.60, *df* = 14, *P* = 0.556; C: *t* = −0.78, *df* = 14, *P* = 0.450; CG: *t* = −2.29, *df* = 14, *P* = 0.038; F: *t* = 1.88, *df* = 14, *P* = 0.081; FG: *t* = 4.07, *df* = 14, *P* = 0.001).

**Table 1:** Description of baseline values measured before interventions and resting motor threshold (RMT) changes after interventions.

	MVC	MEP amplitude (mV)	Stimulus intensity (%MSO)	RMT (intensity, %MSO)	
	EMG (mV)			Baseline	30 min
PAS25	—	0.74 ± 0.07	68.4 ± 3.8	49.9 ± 2.6	50.4 ± 2.4
C	5.64 ± 0.40	0.85 ± 0.04	68.0 ± 3.6	50.1 ± 2.5	50.5 ± 2.5
CG	6.19 ± 0.40	0.80 ± 0.04	68.7 ± 3.5	50.1 ± 2.6	48.8 ± 2.6
F	5.89 ± 0.43	0.75 ± 0.04	68.1 ± 3.1	50.2 ± 2.7	48.5 ± 2.3
FG	6.42 ± 0.41	0.66 ± 0.03	71.7 ± 3.6	49.0 ± 2.8	46.0 ± 2.5 **

MSO, maximum stimulus output. The maximum output intensity of the TMS apparatus used. **P < 0.01.

### Behavioral Data

**Figure 2:**
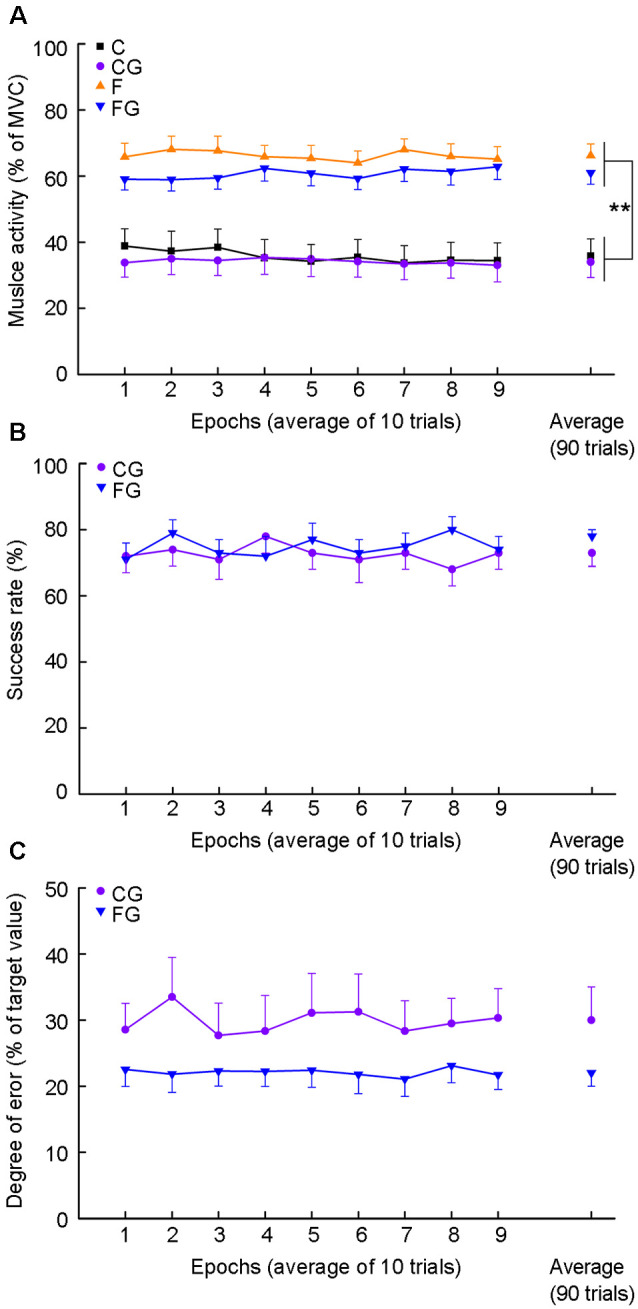
Muscle activity, success rate, and degree of error for motor tasks. Data obtained from 15 subjects. **(A)** Average muscle activity for four motor tasks. The abscissa indicates nine epochs by binning 10 consecutive movements and average muscle activity (right plots). The ordinate indicates muscle activity expressed as average peak EMG activity normalized as a percentage of maximum voluntary contraction (MVC). **(B)** The success rate for two controlling tasks. The ordinate indicates the success rate. **(C)** Degree of error for two controlling tasks. The ordinate indicates the degree of error. The degree of error was calculated as the absolute vertical distance from the peak EMG activity of each trial to the target center, and normalized as a percentage of the values of the target center, i.e., mean of the C in the CG and mean of the F in the FG. **P < 0.01, comfortable muscle activity vs. forceful muscle activity.

Figure 2 shows changes in peak muscle activity along the ninety trials in four motor tasks (Figure 2A), as well as success rate (Figure 2B) and degree of error (Figure 2C) in two motor tasks with goals. For time-course changes in muscle activity, there was no significant main effect in time for four motor tasks (C: *F*_(3.08,43.15)_ = 1.26, *P* = 0.272; CG: *F*_(8,112)_ = 0.76, *P* = 0.640; F: *F*_(3.59,50.20)_ = 0.97, *P* = 0.463; FG: *F*_(3.81,53.35)_ = 1.86, *P* = 0.134). Additionally, no significant interaction effect between muscle activity and goal-setting was found among the average of four motor tasks (*F*_(1,14)_ = 0.66, *P* = 0.431). The main effect was significant for muscle activity but not for goal setting (muscle activity: *F*_(1,14)_ = 21.68, *P* < 0.001; goal-setting: *F*_(1,14)_ = 4.31, *P* = 0.057; Figure 2A, right plots).

There was no significant main effect in 9 epochs for each goal-setting task in success rate (CG: *F*_(4.01,56.18)_ = 0.56, *P* = 0.0.812; FG: *F*_(8,112)_ = 0.71, *P* = 0.679; Figure 2B) and degree of error (CG: *F*_(8,112)_ = 0.67, *P* = 0.714; FG: *F*_(8,112)_ = 0.22, *P* = 0.987; Figure 2C). Additionally, no significant difference was found in average of success rate or degree of error between CG and FG (success rate: *t* = −1.40, *df* = 14, *P* = 0.182; degree of error: *t* = −1.95, *df* = 14, *P* = 0.071; Figures 2B,C, right plots).

### MEP Data

**Figure 3:**
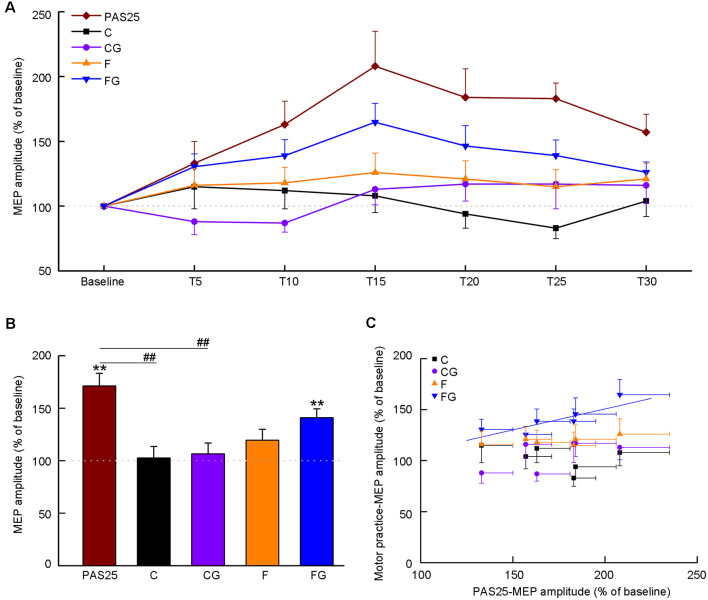
Effects of interventions on motor-evoked potential (MEP) amplitude. Data obtained from 15 subjects. The coordinate indicates the MEP amplitude normalized as a percentage value to baseline tested before the intervention. **(A)** Time course of MEP amplitude before and after five interventions. The abscissa indicates the time from baseline to 30 min after interventions. **(B)** Average MEP amplitude after interventions. **(C)** Correlation between PAS25 and motor tasks. The abscissa indicates the average MEP amplitude at six-time points (5, 10, 15, 20, 25, 30 min) after PAS25. Solid lines represent the significant correlation between normalized MEPs only after PAS25 and FG. All the symbols are mean ± SD for both *x* and *y* values. **P < 0.01, compared with baseline (100%). ^##^*P* < 0.01, PAS25 vs. C/CG.

We investigated the temporary changes (from baseline to 30 min) in MEP amplitude for each task (Figure 3A). The results are shown as individual data (Supplementary Figure 1) and average data (Figure 3A), respectively. The Friedman test detected significant effects only in PAS25 and FG (PAS25: *χ*^2^ = 26.35, *df* = 6, *P* < 0.001; C: *χ*^2^ = 7.20, *df* = 6, *P* = 0.303; CG: *χ*^2^ = 11.69, *df* = 6, *P* = 0.069; F: *χ*^2^ = 2.49, *df* = 6, *P* = 0.870; FG: *χ*^2^ = 21.74, *df* = 6, *P* = 0.001). *Post hoc* analysis with the Wilcoxon test confirmed that PAS25 showed a significant increase in MEP amplitude from T10 to T30 compared with baseline (compared to 100%, significance threshold was set at *P* < 0.008, i.e., 0.05 ÷ 6 time points after interventions ≈0.008. T10: *Z* = −3.01, *P* = 0.003; T15: *Z* = −3.35, *P* = 0.001; T20: *Z* = −3.05, *P* = 0.002; T25: *Z* = −3.41, *P* = 0.001; T30: *Z* = −2.95, *P* = 0.003), while FG showed a significant increase in MEP amplitude from T10 to T20 compared with baseline (compared to 100%, significance threshold was set at *P* < 0.008, i.e., 0.05 ÷ 6 time points after interventions ≈0.008. T10: *Z* = −3.35, *P* = 0.001; T15: *Z* = −3.41, *P* = 0.001; T20: *Z* = −2.73, *P* = 0.006).

Figure 3B shows the averaged MEP amplitude in the post-intervention phase (5–30 min) for five interventions. One-way ANOVA detected a significant main effect among the five interventions (*F*_(4,56)_ = 7.72, *P* < 0.001). *Post hoc* analysis confirmed that the MEP amplitude of PAS25 was significantly higher than that of C and CG (PAS25 vs. C: *P* = 0.009; PAS25 vs. CG: *P* < 0.001; PAS25 vs. F: *P* = 0.111; PAS25 vs. FG: *P* = 0.780). We also analyzed the changes in MEP amplitude for each task compared with baseline. MEP amplitude after the intervention was bigger than that of the baseline only for PAS25 and FG (compared to 100%, Bonferroni’s correction, significance threshold was set at *P* < 0.01, i.e., 0.05 ÷ 5 interventions = 0.01. PAS25: *t* = 5.79, *df* = 14, *P* < 0.001; C: *t* = 0.24, *df* = 14, *P* = 0.817; CG: *t* = 0.63, *df* = 14, *P* = 0.540; F: *t* = 1.87, *df* = 14, *P* = 0.080; FG: *t* = 4.83, *df* = 14, *P* < 0.001). Figure 3C shows the relationship between averaged MEP amplitudes among six post-intervention time points (T5–T30) in the PAS and in each motor task. Significant positive correlation was found only between PAS25 and FG [C: *F*_(1,5)_ = 1.04, *r* = 0.453, *P* = 0.367; CG: *F*_(1,5)_ = 2.84, *r* = 0.644, *P* = 0.167; F: *F*_(1,5)_ = 2.61, *r* = 0.628, *P* = 0.182; FG: *F*_(1,5)_ = 11.70, *r* = 0.863, *P* = 0.027, the linear regression equation was *y* = 0.41 × + 0.69].

## Discussion

In the present study, we analyzed changes in CST plasticity after different motor tasks in humans, especially focusing on muscle activity level and the presence/absence of a goal of keeping the activity level constant. Among the four tasks (C, CG, F, and FG), only the FG, which includes both forceful muscle activity and the goal of keeping the activity level constant, elicited an increase in MEP amplitude as compared with the baseline value. However, before concluding that the combination of the two factors is required for a plastic change in CST excitability after motor practice, we must determine that other factors did not affect the results.

We set the experimental paradigm to keep the intervention parameters as consistent as possible among different tasks. Subjects were asked to stare at a computer display during all intervention processes, even in the PAS25, C, and F, to maintain visual stimulation on the sensorimotor network constant (Taylor-Clarke et al., 2002). The total number of times and the frequency of each motor task were also set to be the same as the PAS25 (90 times, 0.2 Hz). In previous studies, the total number of times or frequency of voluntary movement was often higher than those used in the PAS protocol (Rosenkranz et al., 2007; Delvendahl et al., 2012). In the two tasks with a clear goal (CG and FG) in which subjects controlled the muscle activity level, the subjects were made to directly observe and control peak EMG activity using visual feedback. No significant difference was found in RMT before the intervention among the four tasks and PAS25, indicating that the most active group of corticospinal neurons with the lowest firing threshold was not altered among the days when experiments using different interventions were conducted (Hallett, 2007). However, RMT was significantly decreased only at 30 min after the FG. In our study, RMT was not measured immediately after the interventions for the following reasons: (1) it would take too long to measure RMT (about 5 min), which would have made it difficult for us to obtain the MEP amplitude at fixed time points (starting at 5 min after intervention); and (2) as reported by Delvendahl et al. (2012), the changes in RMT would be most prominent 30–60 min after motor practice.

Most previous studies recorded mechanical parameters such as force or acceleration to evaluate the performance of motor tasks (Classen et al., 1998; Ziemann et al., 2004; Rosenkranz et al., 2007; Delvendahl et al., 2012). In the present study, only EMG was recorded and subjects monitored their own peak amplitudes. However, peak amplitude constancy throughout task execution might not necessarily guarantee that there was no change in any kinetic parameters. As supplementary data, we found that the integration of rectified EMG (area under the curve, AUC) was linearly related to the peak amplitude (Supplementary Figure 2). AUC is known to be positively correlated with force (acceleration) in finger movement (Takakura et al., 2010). Thus, the fact that there were no temporal changes in peak amplitude suggests that there would also be no temporal change in force or acceleration.

The increase in MEP amplitude observed after the FG was likely a result of the combined effects of exerting forceful muscle activity and striving for the goal of keeping the activity level constant. The combination of the two factors was not reported as the necessary condition for the increase in MEP amplitude in previous studies. First, much evidence exists that strong voluntary contractions alone can modulate CST excitability (Taylor et al., 1997; Perez and Cohen, 2009). For example, the right wrist isometric force generation with 70% MVC increased MEP amplitude (Perez and Cohen, 2009). Our result is not in agreement with this: MEP amplitude was not increased after the F, but it was increased after the FG. This contradiction could be explained by the difference in the experimental protocol. In the previous studies, a range (or target zone) within which muscle strength should be controlled was given to guide subjects to contract with a specified force intensity (Taylor et al., 1997; Stefan et al., 2006; Perez and Cohen, 2009). This is the same as the experimental protocol of the FG in this study.

The fact that there was no temporary change in the success rate or degree of error in the CG and FG (Figures 2B,C) indicates that subjects did not learn how to control their muscle activity in a target range throughout the 90 trials of each task. Controlling the peak EMG activity of short-lasting ballistic movement might be too difficult for subjects to learn. Even though no motor learning occurred, an increase in MEP amplitude was still observed after the FG. Most importantly, the subjects strove to control the level of muscle activity in the FG. Thus, successfully learning a new movement is not always necessary for changes in MEP amplitude. Rather, repetitively trying to learn a task is, in itself, important. To the best of our knowledge, there has been no report of this kind of plasticity. This does not mean that successful learning is not important for increasing the MEP amplitude. Stefan et al. (2006) showed a significant increase in MEP amplitude after subjects did 15 min ballistic movements with a controlling force of 30–40% MVC, a protocol very similar to the CG in our study. Interestingly, their study subjects improved their performance of the task (keeping the force in the target range), while our subjects did not show any learning. Thus, “learning” could also be an important factor for inducing the increase in MEP amplitude.

The consideration above suggests that many factors influence the change in the MEP amplitude, i.e., (1) muscle activity level; (2) setting a clear goal of keeping the activity level constant; (3) learning; and (4) trying to learn. Especially, we now must take both the effects of learning and those of trying to learn into account. The present study could provide a new experimental model for inducing plasticity using a combination of muscle activity levels with/without setting a goal. The details of how each of these factors and their combined influence the MEP amplitude should be further analyzed.

Since LTP is known as one mechanism of motor learning plasticity (Classen et al., 1998; Ziemann et al., 2004), we wonder whether there is a similarity between the plasticity observed after FG and after PAS25. In the present study, we tested the change in MEP after PAS25 as well as after four motor tasks, on the assumption that if there were a correlation between the MEP increases after the PAS25 and after a motor task, the latter would be associated partly with LTP-like plasticity just as in PAS25 (Stefan et al., 2002). As shown in Figures 3A,B, the MEP amplitudes after both PAS25 and the FG significantly increased as compared with baseline, peaking at 15 min after the intervention. Indeed, there was a significant positive correlation between them (Figure 3C). The increase in the MEP amplitude after the FG would likely be induced with LTP-like plasticity in the M1, repeatedly reported to occur in motor learning (Vallence et al., 2013), at least partially. For this point, although MEP amplitude reflects CST excitability, the LTP-like plasticity by FG might occur in the synapses not only on corticospinal neurons themselves but also on cortico-cortical interneurons. Cortico-cortical synapses might also indirectly activate corticospinal neurons, thus modulating the property of horizontal pathways in the M1, which have been shown to have a capacity for long-lasting synaptic modification (Sanes and Donoghue, 2000).

There was also a difference in the effects of PAS25 and FG on MEP, in that RMT was reduced 30 min after FG but not after PAS25. RMT is thought to reflect the membrane excitability of the intracortical axons in the M1 targeted by TMS (Delvendahl et al., 2012). No change in RMT after PAS25 was also demonstrated in previous studies (Stefan et al., 2000; Sale et al., 2007). LTP-like plasticity induced by PAS25 mainly depended on the activation of NMDA receptors and did not change the membrane excitability (Stefan et al., 2000; Sale et al., 2007). Therefore, changes in RMT after FG suggest that plasticity induced by FG would be associated not only with LTP-like plasticity in the corticospinal system but also with the membrane depolarization of M1 neurons, presumably the corticospinal neurons, through the modulation of voltage-gated ion channels in the axosomatic membrane of the postsynaptic neurons (Zhang and Linden, 2003).

There are some limitations to our study, and future directions are suggested. First, since we started to measure the changes in MEP amplitude within 5 min (T5) after motor tasks, we did not directly assess the possibility of fatigue, because we did not test indexes such as the change in MVC after the tasks. However, it should be noted that there is no change in muscle activity in no-goal motor tasks, i.e., C and F, which suggests that there was no decrease in muscle activity even at the later phase of the no-goal motor task. Also, some previous studies using similar motor tasks, such as 10–60 min ballistic thumb abduction at 0.5 Hz or 0.25 Hz, did not report fatigue effects (Rosenkranz et al., 2007; Jung and Ziemann, 2009; Delvendahl et al., 2011). The frequency (0.2 Hz) and duration (7.5 min) in our tasks were lower and shorter than those in previous studies. Thus, the subjects in our experiment were not fatigued. Second, the order of interventions was not completely randomized. We tested PAS25 first in all subjects. Then, since the target range of the CG and FG were defined by the result of the C and F, the C was always conducted before the CG, and the F was always conducted before the FG. To avoid an interaction effect of non-random order of tasks, at least 1 week elapsed between any two tasks. Also, the order of the C and F were randomized. A previous study suggested that the impact of PAS25 generally lasts 90–120 min (Wischnewski and Schutter, 2016). Thus, the effect of non-random order of tasks, if any, would be minimum. Third, the results in MEP amplitude measured with ten TMS trials at each time point might be considered to lack stability and reliability since a previous study reported that approximately 20–30 trials were required to provide a stable measure of MEP amplitude with high within- and between-session reliability (Goldsworthy et al., 2016). This issue should be analyzed in detail in future studies. Fourth, since the PAS25 protocol has been reported to increase MEP amplitude more strongly compared with a variety of other NIBS techniques, such as high-frequency rTMS and TBS (Di Lazzaro et al., 2011), we only used PAS25 to induce LTP-like plasticity. However, a combination of different NIBS techniques could be utilized to elucidate the mechanisms of FG-induced plasticity at subcellular levels. Finally, the LTP-like plasticity after FG should be further tested with a metaplasticity paradigm, a combination of PAS25 and motor tasks (Ziemann et al., 2004), which could verify whether the changes in plasticity after FG share the same neuronal network with the LTP-like plasticity induced by PAS25. Other spinal or subspinal level measurements, such as spinal excitability or M wave, would also help to elucidate the physiological mechanisms underlying motor cortical plasticity after FG.

In summary, an increase in MEP amplitude and a decrease in RMT was observed after the FG, short-term motor practice with forceful muscle activity levels plus the requirement of controlling the activity level, although there was no learning throughout the practice. The change in the MEP amplitude correlated with that after the PAS25, in which LTP-like plasticity in the M1 was involved. The change in RMT only after the FG suggests that the membrane depolarization of M1 neurons was also involved in FG. This insight could guide motor rehabilitation from neurological diseases such as stroke and traumatic brain injury: in physical rehabilitation, choosing higher intensity exercise at a level that is acceptable to the patient and setting specific goals can help to promote a change in the excitability of corticospinal pathways.

## Data Availability Statement

The raw data supporting the conclusions of this article will be made available by the authors, without undue reservation.

## Ethics Statement

The studies involving human participants were reviewed and approved by Human Research Ethics Committee of Waseda University (2018-040). The patients/participants provided their written informed consent to participate in this study.

## Author Contributions

WD: conceptualization, methodology, investigation, data analysis, visualization, writing—original draft, writing—review and editing. KN: conceptualization, data analysis, writing—review and editing. TN: writing—review and editing. KK: conceptualization, methodology, project administration, supervision, writing—review and editing.

## Conflict of Interest

The authors declare that the research was conducted in the absence of any commercial or financial relationships that could be construed as a potential conflict of interest.

## Abbreviations

APB: abductor pollicis brevis
AUC: area under the curve
C: comfortable task
CG: comfortable with a goal task
CST: corticospinal tract
EMG: electromyogram
F: forceful task
FG: forceful with a goal task
LTP: long-term potentiation
M1: primary motor cortex
MEP: motor-evoked potential
MVC: maximum voluntary contraction
NIBS: non-invasive brain stimulation
PAS: paired associative stimulation
RMT: resting motor threshold
TMS: transcranial magnetic stimulation.
