# Supplementary material for: Determinants of Neural Plastic Changes Induced by Motor Practice
Source: Front Hum Neurosci. 2021 Jan 28;15:613867. doi: 10.3389/fnhum.2021.613867 (PMC7875877; doi:10.3389/fnhum.2021.613867)

Supplementary materials

**SUPPLEMENTARY METHOD**

*Statistics*

We calculated the area under the curve (AUC) of muscle activity for each motor task. The AUC onset and offset for each trial were calculated by ten standard deviations with electromyographic data after rectifying and band-pass filtering between 55 Hz-450Hz 1s before the first tone.

A linear regression analysis was performed to evaluate the relationship between the peak amplitude and AUC of muscle activity in all four motor tasks for 15 subjects. Before the linear regression analysis, the Smirnov-Grubbs' test was used to check outliers in AUC.

**SUPPLEMENTARY RESULT**

Supplementary Figure 1 shows the individual data (15 subjects, No. 01, No. 02 … No. 15) for the temporary changes (from baseline to 30 min) of MEP amplitude for five tasks.

Supplementary Figure 2 shows a significant positive correlation between the peak amplitude and AUC in muscle activity in all four motor tasks for 15 subjects with one outlier removed (F(1, 58)=69.44, r=0.74, P<0.001). We eliminated one outlier from the correlation analysis using the test of rejection of Smirnoff-Grubbs (T=3.92, P=0.002). The linear regression equation was y = 0.20x - 0.02).

**Supplementary Figure 1**


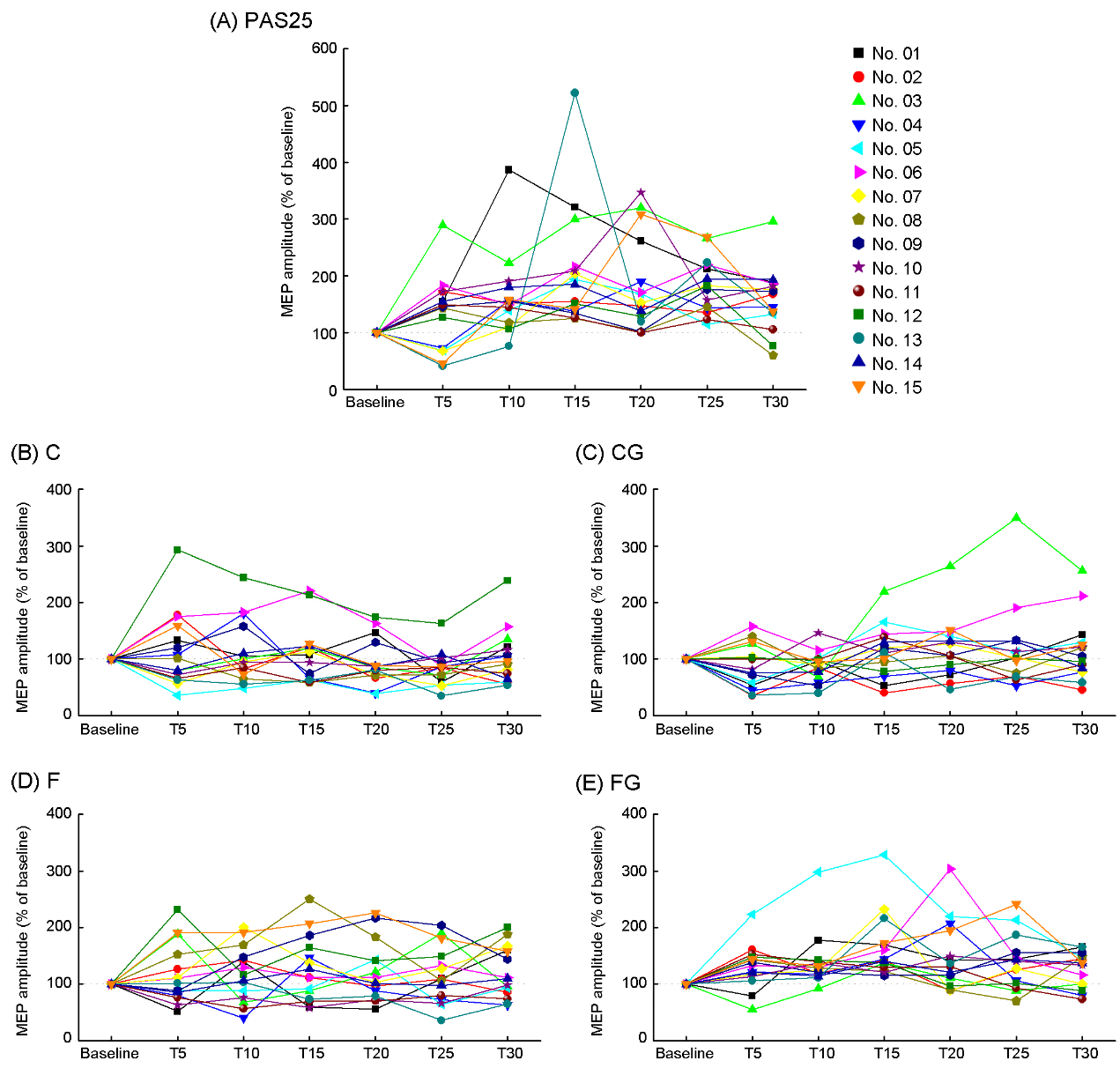


**Supplementary Figure 2**


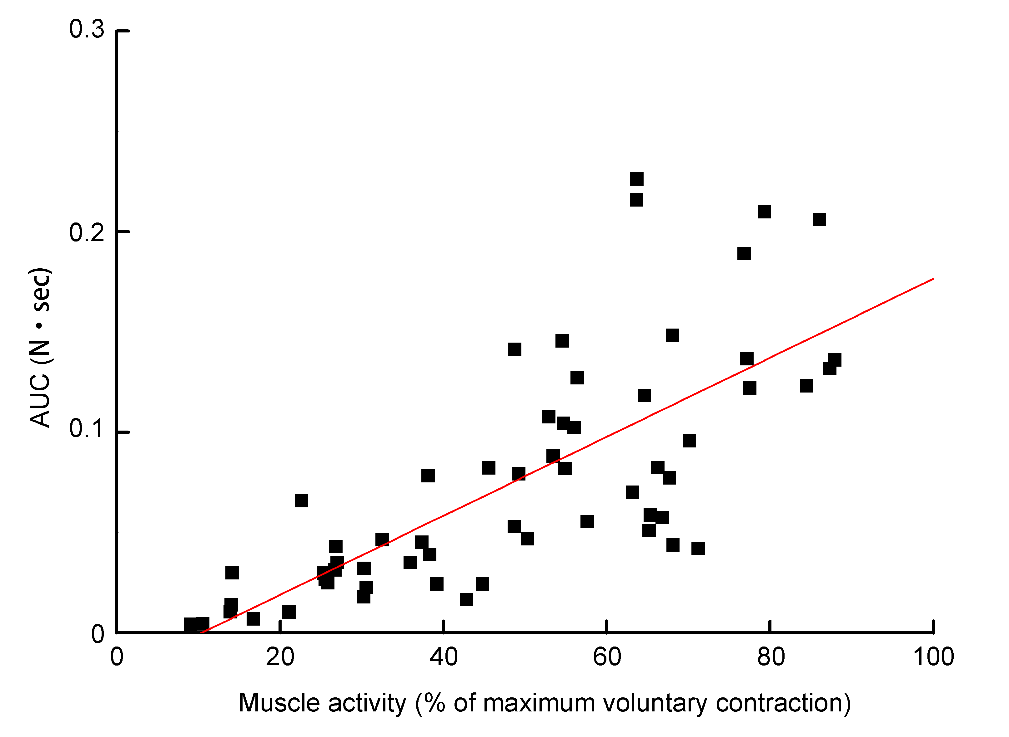

Supplement: Supplementary file 1 [file Table_1.docx]
